# Tackling AMR from a multidisciplinary perspective: a primer from education and psychology

**DOI:** 10.1007/s10123-022-00278-1

**Published:** 2022-10-13

**Authors:** Alicia Calvo-Villamañán, Álvaro San Millán, Laura Carrilero

**Affiliations:** 1 Department of Microbial Biotechnology, Centro Nacional de Biotecnología–CSIC, 28049 Madrid, Spain; 2 School of Biosciences, The University of Sheffield, Western Bank, Sheffield, S10 2TN UK

**Keywords:** Antimicrobial resistance, Antibiotic resistance trend, Educational psychology

## Abstract

Antimicrobial resistance (AMR) is currently one of the most concerning threats in public health. The efforts to tackle the problem require a global One Health approach, using multidisciplinary approaches and a thorough understanding of the topic both by the general public and the experts. Currently, the lack of a shared mental model of the problem, the absence of a sense of responsibility amongst the different actors and a deficient education on the topic burden the efforts to slow down the emergency and spread of antimicrobial resistant infections. We here propose a multidisciplinary approach to tackle the AMR problem, taking into consideration not only the input from the biological and medical sciences but also the input from the social sciences. Specifically, we suggest strategies from education and psychology to increase awareness about antimicrobial resistance and to implement more effective interventions. Finally, we advocate for a comprehensive and a solidaristic model as the only solution for a problem which knows no borders. As such, political will and international cooperation will be key to achieve the desired change in antibiotic resistance trend.

## Introduction

Antimicrobial resistance (AMR) is widely considered to be the next global pandemic that humanity is going to face. In fact, many consider this pandemic to be already on-going, with the world-wide number of estimated deaths by AMR elevating to almost 5 M in 2019 (Murray et al. 2019). The World Health Organisation (WHO) has labelled AMR as one of the top 10 global public health threats, equating AMR to the threat level of HIV, dengue, COVID-19 or future influenza pandemics (Ten health issues WHO will tackle this year, 2019; 10 global health issues to track in 2021).

Studies have shown that by 2050, AMR could lead to a decrease of the annual GDP of roughly 1%, with this value being as high as 5–7% in developing countries. This would translate to world-wide losses of 100–200-trillion € (World Bank 2017), further widening of the gap between rich and poor. These values might be underestimated, as the clinical AMR problem has been deepened by the generalised preventive use of antibiotics in hospitalised COVID-19 patients worldwide, which might have accelerated the problem and whose impact is still not understood today.

Nowadays, one of the main concerns for AMR is the clinical setting. The European Centre for Disease Prevention and Control (ECDC) estimates that almost 9 million nosocomial infections (or healthcare-associated infections) occur each year in European hospitals alone, with ${~}^{1}\!\left/ \!{~}_{3}\right.$ of these infections being caused by bacteria with some level of AMR (Plachouras et al. 2018; Suetens et al. 2018; Anderson et al. 2019).

However, AMR is far from being exclusively a healthcare-related problem. The WHO’s list of main drivers for AMR include the misuse and overuse of antimicrobials in both the clinical and farm context, the lack of access to clear water, bad practises in the sanitation and hygiene of both humans and animals, poor access to early diagnostics, a lack of legislation on the matter and last, but definitely not least, a generalised lack of awareness and knowledge on the subject. The AMR crisis is therefore a great example of a problem that should be approached under the holistic perspective of “One Health.”

One Health is the understanding that in today’s globalised world, there is no real distinction between human health, animal health and environmental health. A formal definition of One Health, as provided by the One Health Initiative Task Force of the American Veterinary Medical Association, would be “the collaborative efforts of multiple disciplines working locally, nationally, and globally, to attain optimal health for people, animals and our environment.”

## A shared mental model of AMR

For years, the approach to fighting multidrug resistant organisms (MDROs) has been an almost exclusively scientific one. As AMR started spreading, a huge effort was placed in the search for new antibiotics and the development of new therapies. Although great advances are being made in these domains, it is clear that, given the planetary scale of the problem, a solely wet-lab approach no longer suffices. We believe that such a complex threat requires a multidisciplinary approach, and that the contribution of both biological and social sciences is necessary to address it.

In fact, to fully understand the scale of the AMR problem, one has to take into consideration social disciplines such as economy and politics. On first sight, the link between these disciplines and AMR might seem implausible. However, simple decisions such as, for example, the choice of antibiotic to treat an infection are often made on the basis of cost-effectiveness, which is above all, an economical decision. Given that treatment guidelines are usually set by national public health bodies, this often creates a uniform national strategy against infection, meaning that the same antibiotic will be often used throughout an entire territory (Wagner 2020; Laxminarayan and Weitzman 2002). This creates a perfect setup for the rise of AMR, as it is known the use of a single drug promotes the evolution of AMR (Santos-Lopez et al. 2019). A solution to this problem could be to informedly prescribe different antibiotics to different patients, with the aim of minimising the selection pressure against a single antibiotic. However, different antibiotics have radically different prices, and therefore, this would only be possible if policy interventions were set in place to adjust the price of the different antibiotics (Laxminarayan and Weitzman 2002). Therefore, even disciplines as far from biology as economy and politics play a role in the day-to-day spreading of AMR.

**Fig. 1:**
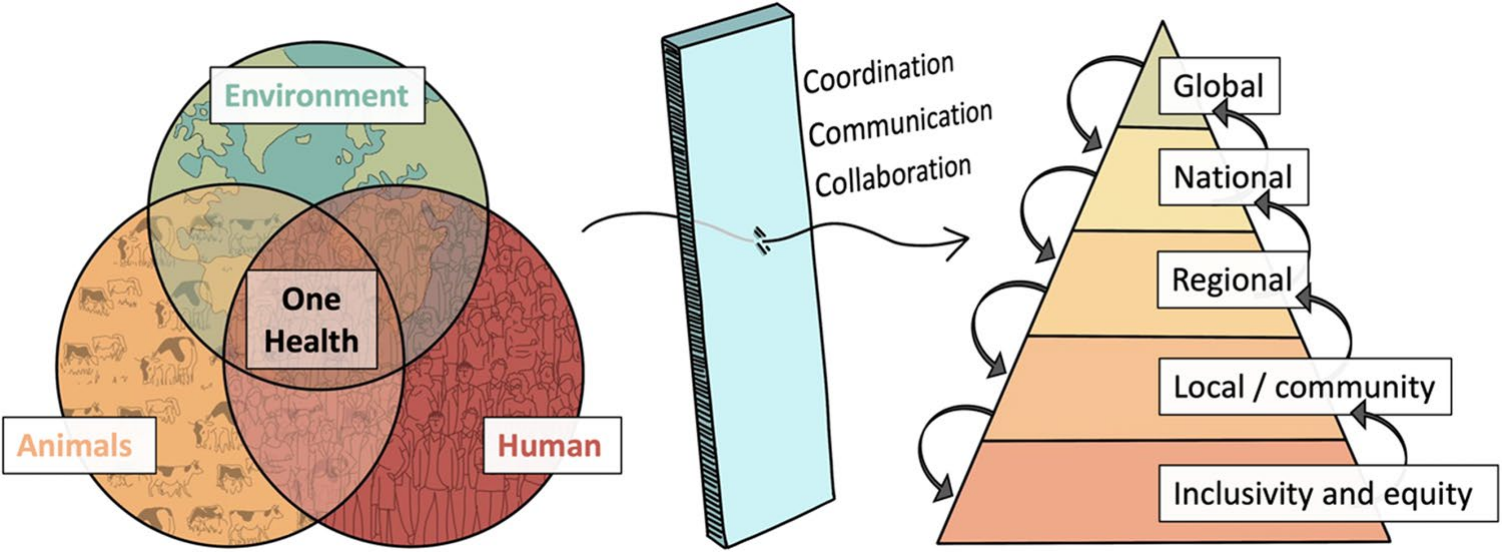
A suitable framework to tackle AMR requires approaching the problem from a One Health perspective, acting at the global, national, regional and local levels. Only through efforts of coordination, communication and collaboration between all different levels of organisation would such approach be effective

It is clear that promoting collaboration between radically different disciplines is an essential step in the fight against AMR (Fig. 1). One first step in promoting such collaboration is to set a common ground, what is known as a shared mental model. Mental models are essential in the process of reasoning, as they are the individual’s internal representation of an external reality. They are therefore essential to understand the way in which each individual interacts with their reality.

Having an accurate and shared mental model of AMR drivers and the different factors that play a role in the problem, in addition to the diverse knowledge areas that can contribute to preserving the usefulness of antibiotics, would promote conversations and facilitate the necessary investment from the different concerned parties. Additionally, the COVID-19 pandemic has highlighted the urgency of developing global policies and legal frameworks to deal with health crises that in a global society know no borders. The same will be true for the AMR pandemic in the near future.

The COVID-19 crisis has also highlighted that a poorly scientifically educated population will be less likely to understand the logic behind health policies, and will therefore be less likely to abide by them. Taking this in consideration, the data on the general public’s understanding of the use of antibiotics and AMR paints a dark picture. Several studies (McNulty et al. 2007; McCullough et al. 2016; Oniciuc et al. (2019)) have revealed a generalised lack of knowledge on the matter, which can be divided into four categories: (i) a misunderstanding of the concept of AMR itself, (ii) a lack of awareness of the possibility of infection by MDROs, (iii) a lack of awareness of one’s individual role in the AMR problem and (iv) a misunderstanding of how antibiotics work. They also revealed that understanding of the topic is strongly linked to education level, and that exposure to primary information can lead to appropriate behavioural changes (Tsuzuki et al. 2020).

Studies that looked at the different groups involved in antibiotic use in separate (general practitioners, hospital physicians, veterinarians, farmers and the general public) reveal that all groups tended to believe that the main drivers of AMR came from the other groups (Schneider et al. 2018). This phenomenon is known as *playing the blame game*. Three interesting facts arise from this observation. First, it reveals a lack of understanding of the individual responsibility on AMR spread, which undoubtedly plays a role in the speed at which it takes place. Secondly, when people play the blame game, they often engage in further irrational thinking in order to justify blaming others. This makes it even harder for fundamental changes to be made in society. And thirdly, this indicated the lack of a shared mental model on AMR by the different groups involved in its spread.

A shared mental model about AMR would therefore require informing each actor about their responsibility and confronting their misbeliefs on their lack of individual responsibility on the AMR problem. In order to achieve it, we need to change the way the different groups interact with the problem. One way to do this would be to apply bottom-up processing techniques within each group. In simple terms, bottom-up processing requires understanding the current practices in the community and the reasons behind them as a starting point to address the AMR challenge. Adopting this framework would be particularly relevant in contexts where there is often limited input from health professionals, such as in the case of developing countries (Caudell et al. 2020).

By empowering the general population and promoting social responsibility amongst it, situations such as the overprescription of antibiotics could also be slowed down. This is particularly important in light of a recent systematic review that showed that even when confronted with the link between overprescribing and AMR, prescribers considered it futile to change their prescription behaviour (Krockow 2020).

## Finding suitable frameworks to design effective interventions to tackle AMR

Adopting a shared mental model about AMR would therefore facilitate a global change in behaviour. This is not a simple task, as behaviour determinants are complex, and usually interventions aiming at behavioural change need to be contextualised and adapted to each of their target groups. A comprehensive approach to promote behavioural change would require diagnosing the factors influencing the antibiotic use, implementing the best interventions on the light of the diagnosis and evaluating the outcomes both quantitatively and qualitatively. Amongst others, perceptions, attitudes, knowledge, pressures, norms (social and personal), values (social and personal) and worldviews, these are all factors that influence antibiotic prescribing behaviour and/or use, and they are bound to be very different amongst different people.

Efforts have been made in the direction of providing a suitable framework that accounts for the complexity of antibiotic prescription and use. One example of such frameworks that has been proposed is the behaviour change wheel (Michie et al. 2011; Flowers 2018), a general method to design behaviour change interventions. This is a method that allows for the characterisation and design of behaviour change interventions. The framework takes into account three conditions necessary for a behaviour to occur: (a) capability, the capacity (physical and psychological) to engage in the activity, including the knowledge and the skills needed; (b) motivation, the mental processes that direct behaviour, including emotional responding and analytical decision making; and (c) opportunity, the factors outside of the individual that make the behaviour possible. This framework allows one to tailor the best intervention for each specific context, maximising the outcomes. For example, in long-term care facilities, where the level of inappropriate prescribing has been identified as high as 75%, the application of the behavioural change wheel has identified the incorporation of environmental restructuring and performance feedback as promising intervention strategies to promote more mindful prescribing (Crayton et al. 2020).

The desired radical change of behaviour would benefit from a community that strongly understands the policies set in place. It has been shown that lack of knowledge is one of the main drivers of irrational use of antibiotics (both by the general public and by healthcare providers (Machowska and Lundborg 2018)). It is important to highlight, however, that a higher level of knowledge alone does not necessarily lead to a more appropriate antibiotic use (Cantarero-Arévalo et al. 2017; Pham-Duc et al. 2019).

Taking everything into consideration, and given the global context, it is clear that efforts need to be made to increase the understanding of the general population of the AMR problem, especially in situations in which the lack of knowledge and understanding is the main barrier for a better antibiotic use. It will be particularly important to integrate the individual selfish approach to health into local and global One Health actions. In other words, it is vital to confront individualistic perspectives with the need for a collective responsibility that will undoubtedly enter in direct confrontation with individual freedoms (Hernando-Amado et al. 2020). One way of achieving this could be through educational campaigns.

## The complex role of healthcare in AMR spread

In the past decades, several countries have launched campaigns targeting hospital physicians, as they play a crucial role in the problem when wrongly prescribing antibiotics (McNulty et al. 2012; Helou et al. 2020). It has been suggested that clinicians should play an active role in the education of patients, informing of the risks of acquisition of resistant bacteria (Crayton et al. 2020).

In fact, a common motivation for the prescription of antibiotics is wanting to keep a good relationship with the ill-informed patient (Gonzalez-Gonzalez et al. 2015). Because of this factor, patient education by doctors is key, playing an active role in their understanding of AMR. In fact, a study showed that when the parents of sick children are provided with proper explanations about the lack of need for antibiotics (e.g. for a common cold), they were less likely to be disappointed by the lack of prescription of antibiotics (Cantarero-Arévalo et al. 2017). Therefore, prescriber’s education regarding social skills would help them manage patient’s expectations, to build trust and to educate their clients without sacrificing a good doctor-patient relationship if antibiotics are not prescribed.

Conversely, it is essential to specifically train healthcare workers on the topic of AMR. In fact, several institutions have started offering multidisciplinary programs to train physicians on the use of antibiotics, using teams comprising pharmacists, epidemiologists and microbiologists (Lee et al. 2015). A few campaigns have targeted health students, as they have been shown to feel the need for more education on the topic (Dyar et al. 2018). For example, in the UK, a set of materials for prudent antimicrobial prescribing was developed for medical students (Pulcini and Gyssens 2013) (PAUSE http://www.pause-online.org.uk).

When educating healthcare workers on the topic, it is important to tailor each campaign to each individual’s context. A review showed that in developing countries, healthcare workers with a good knowledge of AMR often lacked information about local AMR levels and profiles (Chaw et al. 2018). In this case, providing up-to-date information on local epidemiology of AMR could be the most efficient way of circumventing the lack of diagnosis.

It has also been shown that the dynamics amongst different generations of healthcare workers have a strong impact on the adoption of measures to slow the spread of AMR. These dynamics determine whether younger doctors decide to follow senior clinicians’ prescribing habits or the consideration of aids such as proposed guidelines (Papoutsi et al. 2017). Promoting a culture of continuous development amongst health practitioners and getting prescribers involved in the development of measurements could help to align guidelines and real clinical practices, thus avoiding the resistance that changes in policies can encounter.

Other campaigns have targeted patients, trying to reduce the demand for antibiotics. This is necessary because there is a known expectation from health service users for antibiotic prescription. A second strong issue regarding this reality is that individuals tend to assess their personal immediate risk of resistance-related effects to be low. Therefore, a useful type of intervention on patients would be to emphasise the non-negligible possibility of the serious side effects of using antibiotics (Klein et al. 2018; Brooks et al. 2008). This strategy has proven successful, as one intervention encouraging patients to raise the topic of unnecessary prescriptions to their doctors was shown to reduce the overall antibiotic prescription in primary care (Altiner et al. 2007).

There are several examples of international and national efforts to promote better antibiotic use. For example, the WHO started the “World Antimicrobial Awareness week” back in 2015, an annual effort to encourage better practices in all different groups (World Antimicrobial Awareness Week n.d). Notably, antibiotic awareness day campaigns have been shown to promote a positive trend in behavioural change in Poland, with a highlight on the power of the use of the internet as an educational tool (Mazińska et al. 2017).

In the USA, the CDC started the “Be Antibiotics Aware” campaign (Use and | CDC 2021) and Public Health England launched the “Keep Antibiotics Working” campaign (Keep antibiotics working [overview] n.d). This type of public health campaigns can lead to a more careful use of antibiotics, at least in high-income countries (Huttner et al. 2010). The EU launched the EU-JAMRAI (Joint Action on Antimicrobial Resistance and Healthcare-Associated Infections) which amongst many other things launched the Micro-Combat game to educate on the role that different parties play in AMR spread (https://eu-jamrai.eu/micro-combat/).

## The problem of AMR in farms, the food industry, water and natural ecosystems

Similarly to human health, animal health, especially that of livestock, also plays a crucial role in the spread of AMR. In fact, the overuse of antibiotics in farms far surpasses the overuse in human health, with roughly two-thirds of the antibiotic consumption being destined at livestock (Done et al. 2015). Animal production has historically strongly benefitted from the use of antibiotics, not only for controlling infections but also because antibiotics have been used as growth factors for several species. Because of this, several countries have put regulations in place to reduce the use of antibiotics in farms. For example, the EU prohibited the use of antibiotics as growth promoters in livestock back in 2006, and in 2022 forbid the routine use of antibiotics in farming altogether, including as prophylaxis. This is however not the case in the USA, where around 80% of antibiotics used each year serve non-therapeutic purposes in livestock production (Done et al. 2015). This data indicates the need for educational campaigns directed at farmers and other food-industry professionals. One such campaign is “The Alliance to Save Our Antibiotics,” which is calling for an EU-wide 80% reduction to farm antibiotic use by 2050 (https://www.saveourantibiotics.org).

Apart from human and animal health, another source of preoccupation for the spread of AMR is the contamination of natural ecosystems. MDROs enter natural ecosystems mainly through infected human or animal stools (Karkman et al. 2019). Sewage and water-treatment plants are therefore hotspots for the spread of AMR (Moura et al. 2010). There is a strong need for standardisation of AMR markers in these plants, as well as a global surveillance system of these markers (Hendriksen et al. 2019).

Lastly, the effects of global warming are also likely to have an effect on the spread of AMR. Changes in the behaviour of vector species, such as different bird migration patterns or the colonisation of new environments by flies as temperatures change, are undoubtedly going to affect the global distribution of pathogens. Changes in the temperature of oceans and oceanic currents will likely have a similar effect (Reverter et al. 2020). This data indicates a link between two of the biggest current concerns of humanity: AMR and climate change, and highlights the need for educational campaigns that enforce the idea of collective responsibility (Harring and Krockow 2021).

We recommend the following bibliography to learn more about the topics discussed in this section (Hernando-Amado et al. 2020, 2019).

## Introducing AMR education in school education systems

In the previous sections, we have discussed several existing educational campaigns against AMR. Few of these campaigns, however, have targeted the school educational system. Studies have shown that early childhood is an optimal stage for learning (Institute of Medicine and National Research Council 2015). Our learning capabilities are formed primarily during these years (Melhuish 2014), which means that experiences during this time have profound and long-lasting consequences. Prime examples of initiatives targeting pre-university students in Europe are e-Bug and MicroMundo. E-Bug (https://www.e-bug.eu/) is a European commission-funded effort that provides teaching resources on the use of antibiotics, hygiene and the spread of infection. The program offers several activities tailored for two age groups (9–11 and 12–15 years old), all covering information on how the immune system fights infection, antibiotic use and the importance of vaccination. MicroMundo (Valderrama 2018) is a Spanish and Portuguese initiative involving 20 different universities that aims at teaching high schoolers about AMR by isolating antibiotic producers from soil samples. It is a branch of the international projects Tiny Earth and Small World Initiative. These initiatives have been shown to lead to an improvement in the awareness of AMR in pre-university students (Bueso-Bordils et al. 2020; Maicas et al. 2020). Other examples are the “Do bugs need drugs?” program (https://dobugsneeddrugs.org/), launched by Alberta Health Services and the British Columbia Centre for Disease Control; the debate kit launched by the Spanish National Plan for AMR (https://resistenciaantibioticos.es/en/node/508); or the ReAct Campaign, a set of international independent networks to educate on the nature of AMR and its drivers (https://www.reactgroup.org/). Smaller initiatives have also taken place, such as the “Bugs in Bangkok” board game, which explores the complexity of AMR in great detail (https://bugsinbangkok.wordpress.com/).

The need for the introduction of AMR education in all levels of the educational system follows a trend in recent years of concerns for developing a stronger STEM education. STEM is a term used to refer to all fields of Science (including Health), Technology, Engineering and Maths. The urgency for improving STEM skills is shared by educators, policy developers and industry/business organisations alike, and has its roots mostly on the growing demand for STEM specialists in the labour market and the already existing shortage in the current STEM workforce (Caprile et al. 2015). To these concerns, we would like to add the inevitability of future pandemics in our current setup of a highly globalised humanity and the inevitability of the requirement of STEM specialists to tackle them.

Because of this growing concern for strongly educating future generations, STEM education has been systematically reinforced in the different educational systems. An example of this is the Evolving STEM initiative, aiming at improving the understanding of children of evolution (Cooper et al. 2019). The same is not true for AMR education, where examples of such are scarce (Kvint et al. 2020). If we want to be able to reliably fight the AMR pandemic, we need to begin the systematisation of AMR education.

In order to do so, several facets will have to be evaluated. The first thing that needs discussing is how the topic of AMR will be introduced within the umbrella of STEM integration. The concept of STEM integration defends the idea that regardless of how different STEM fields are between them, the set of skills required for understanding them are very similar (English 2016). Because of this, it is essential to create learning transfer between the different STEM disciplines as they are learnt by the young students (Kelley and Knowles 2016). A project-based learning approach in which knowledge from the different STEM disciplines needs to be applied to the AMR problem could be an appropriate way of promoting STEM integration. For example, learning physical properties of materials and how they could prevent bacterial biofilm formations or using simple mathematical models to understand resistance spread could be part of it, making AMR a transversal topic.

Something else that needs to be assessed are the psychological and sociological determinants of effective education, which would allow us to tailor AMR education to the different publics. Meaningful learning, i.e. completely understanding the learnt information and connecting it with previously known knowledge, requires fitting the new information with one’s existing understanding of the world (Paolo et al. 2014). In order to promote this, framing the learning situation to consider the learner’s values and interest could help to the active processing of information. In the case of formal learning situations, such as schools, giving choices to the pupils, such as writing an essay or doing a craft about a proposed topic, could boost motivation and therefore learning. AMR understanding also implies understanding concepts such as evolution with which there can be a general misunderstanding and all sorts of misconceptions. These cases are particularly complicated, as it has been shown that learners often avoid the cognitive conflict that could lead to conceptual change and real understanding, even when they are confronted with information and facts that challenge their misconceptions (Mayer 2008).

## AMR education for the general public

It has been argued that STEM education, given that it finds itself in the border between science sociology and sociology of education, might suffer from the sociological determinants of both the education and the sciences fields (Xie et al. 2015). This should be taken into consideration when best tailoring AMR education to different publics. For example, it has been shown that role models are very important for the success of STEM education, and that different groups of people need different role models (Gladstone and Cimpian 2021).

It would also be interesting to include gender mainstreaming in AMR education, as AMR will affect women and men differently (Schröder et al. 2016). If we focus on biological sex, women are more likely to suffer from specific infections than men. Urinary tract infections are far more common in women (Harrington and Hooton 2000; Magliano et al. 2012), and childbirth (MacLean 2001), abortion and sanitary healthcare all expose women to higher risks of infection than men. Furthermore, if we focus on gender, female-dominated professions such as healthcare, cleaning and nursing are also more exposed to infection and disease (Office for National Stattistics, UK 2012; World Health Organization 2007; Binagwaho and Mathewos 2022; United Nations Development Group (UNDG)–Western and Central Africa 2015). This means that while men and women share many of the risks posed by AMR, gender plays a role in the likelihood and way of contracting infections, and therefore, the AMR problem has a gender dimension. Therefore, having a gender perspective on AMR education will be essential to provide specific resources and education to an especially vulnerable population (Vooren et al. 2022).

Many other questions will have to be addressed as well, such as at what age to start introducing the topic, or how to better assess the effect of all the different initiatives and campaigns. Psychology approaches could inform the best ways to maximise the effectiveness of interventions. For example, evaluation of policies including qualitative data to understand how individual factors such as values impact the implementation of guidelines could help in the adoption of policy measures (Redding 2020). Tailoring the language and delivery methods of interventions to the target audience and alignment of the message with the values of the target population would without a doubt increase its impact (Redding 2020). For example, while audiences with hierarchical worldviews would support judicious antimicrobial use through increased regulation coming from experts (Broom et al. 2021), audiences with egalitarian worldviews would focus on how everyone could contribute toward the larger goal (Redding 2020), but it would be very difficult to introduce a solidaristic model in the face of individualistic values (Redding 2020).

Drawing upon research on other areas could also inform on how to best tackle the AMR issue. For example, AMR shares several characteristics with climate change, such as its perception of being a slowly emerging problem. Therefore, lessons learnt from public awareness campaigns regarding climate change could be applied to combat AMR.

## Conclusion

All in all, there is no doubt of the urgency of better educating the population and future generations on AMR and how to prevent or slow down this global problem. To effectively tackle AMR, and in line with Broom et al. (Broom et al. 2021), we advocate for a solidaristic model. The nature of AMR requires a global effort in which we all share the responsibility of antibiotic stewardship for a greater social outcome. Big efforts will have to be made which will involve different specialists, institutions and governments, but the examples of AMR education to date can serve as a base and as an example to efforts to be made in the future.

There are several initiatives that could be launched, both at local, national and worldwide levels. Regardless of the scale, one thing to keep in mind is to always tailor the content so that it is relevant to the target public. This is crucial to promote interest and assure that the public has the needed prior knowledge to actively learn about the topic.

Several factors will contribute to the success of any designed campaign. First, the more aligned the campaign is with the hegemonic values of the community, the more effective it will be. To overcome this difficulty, different platforms and styles should be used to reach different target audiences. Second, all different groups, the whole society, should be targeted, in order to reinforce a true change and create a new social norm that is able to endure and create a behavioural change overtime.

Finally, a real political and societal commitment is key, with the will to invest in strategies that will have an impact and a benefit in the long term, and not only focussing on short-term economic goals in healthcare.

Finally, educational changes alone will not suffice. It is crucial that policy plays a crucial role in the fight against AMR, by regulating and directing the measures that are to be taken in order to tackle the problem. In economics, situations in which individuals acting independently according to their own self-interest lead to a depletion in a resource at a community level are referred to as “the tragedy of the commons.” The depletion of the resource is caused by an uncoordinated action at a community level. The tragedy of the commons is usually linked to environmental science and sustainability and it applies perfectly to the AMR problem as well (Cully 2014; Baquero and Campos 2003). To prevent a tragedy of the commons in AMR, we need a global-coordinated response that can only be achieved through regulation.
